# Effect of visual attention and horizontal vergence in three-dimensional space on occurrence of optokinetic nystagmus

**DOI:** 10.16910/jemr.12.1.2

**Published:** 2019-02-28

**Authors:** Kei Kanari, Hirohiko Kaneko

**Affiliations:** Tamagawa University, Tokyo, Japan; Tokyo Institute of Technology, Kanagawa, Japan

**Keywords:** Eye movement, eye tracking, vergence, attention, stereopsis, binocular disparity, optokinetic nystagmus

## Abstract

OKN corresponding to the motion of the fixating area occurs when a stimulus has two areas separated in depth containing motion in different directions. However, when attention and vergence are separately directed to areas with different motions and depths, it remains unclear which property of attention and vergence is prioritized to initiate OKN. In this study, we investigated whether OKN corresponding to motion in the attending or fixating area occurred when two motions with different directions were presented in the central and peripheral visual fields separated in depth. Results show that OKN corresponding to attended motion occurred when observers maintained vergence on the peripheral stimulus and attended to the central stimulus. However, OKN corresponding to each motion in the attending area and in the fixating area occurred when observers maintained vergence on the central stimulus and attended to the peripheral stimulus. The accuracy rate of the target detection task was the lowest in this condition. These results support the idea that motion in the attended area is essential for occurrence of OKN, and vergence and retinal position affect the strength of attention.

## Introduction

The eyes’ rhythmic movement, known as optokinetic nystagmus (OKN), is induced when a sustained moving stimulus is presented in the visual field. OKN consists of a slow phase (pursuit movements in the direction of stimulus motion) and a fast phase (saccadic return movements opposite the direction of motion) [1]. OKN serves to stabilize a moving stimulus’s image on the retina, and it has the following characteristics related to the stimulus’s physical features. OKN gain (ratio of slow phase velocity to stimulus velocity) decreases when a stationary object appears in the plane of the moving stimulus [2]. OKN gain also decreases as the width or area of the moving stimulus decreases [3]. Some studies have reported that OKN gain decreases when the central visual field is occluded [4, 5, 6, 7].

OKN is influenced not only by the stimulus motion at eye position but also by that at attention position, which can be redirected to another location, while eye position is maintained in one location [8]. For example, the motion on which the observer focuses elicits OKN when two patterns moving in different directions are superimposed on the same depth plane [9], and when a motion parallax stimulus containing multiple motion areas with different velocities are presented [10]. Attention paid to motion in the peripheral visual field facilitates OKN corresponding to that evoked by the motion when the central visual field-of-motion stimulus is absent [5, 6]. OKN corresponding to the motion direction of an attended (refers to the attention instructed by the target detection task in this study; the same hereinafter.) stimulus occurs when stimuli moving in different directions are presented in different areas on the same plane [11]. These studies indicate that OKN corresponding to attended motion occurs when stimuli are presented in the two-dimensional plane.

OKN corresponding to a binocularly fused moving stimulus occurs when motion stimuli are presented in different depth planes. For example, when stimuli moving in opposite directions were presented in different depth planes at the central area and its upper and lower areas, OKN corresponding to the binocularly fused moving stimulus occurred [12]. Another study showed that OKN gain decreased as binocular disparity of motion stimulus increased, while vergence was kept on a vertical line with zero disparity relative to the display [13]. Attention seems to affect these results, showing the effect of vergence on OKN because directing vergence to certain depth should involve, at least partly, voluntarily control. However, effects of vergence and attention on OKN in three-dimensional space were not discussed in previous studies.

The results of studies with a two-dimensional stimulus, as shown above, are presumed to show attention’s influence on OKN because the influence of vergence is constant all over the stimulus. Assuming that attention is the essential factor for initiating OKN in three-dimensional space, the claim in the previous study [12] that OKN occurred corresponding to motion on the vergence plane regardless of the central and peripheral visual fields can be interpreted as the effect of the observer’s attention directed with vergence. This study aimed to examine the validity of that presumption by investigating whether OKN corresponding to attended motion occurred when two movements in different directions were separately presented on different depth planes in the central and peripheral visual fields while manipulating vergence distance and attentional state.

## Methods

This study presented two motion stimuli in different directions in the central and peripheral visual fields, separated in depth defined by binocular disparity. The observer attended to one motion stimulus while maintaining vergence distance on the anteroposterior axis at the center of the stimulus, independent of attention location. The observer responded with a numeral presented randomly and moved with the same velocity and direction as the random dots in the attended plane. This task’s purpose was to keep the observer’s attention on the instructed field of stimulus. We verified the vergence state during the trial by measuring binocular eye movements and investigated whether OKN occurred in correspondence to the attention field’s motion or the vergence distance’s motion.

### Participants

One author and six naïve volunteers (six males and one female, aged 23–33 years) participated in this experiment. All had normal or corrected-to-normal visual acuity. They were verified to have a stereo-acuity of at least 40 sec of disparity using a stereo-test (The Fly Stereo-test, Stereo Optical Co., Inc.) and to perceive correctly the stimulus depth with ±4° of horizontal disparity with respect to the display plane before the experiment. All observers provided written informed consent before participating. The study was approved by the Tokyo Institute of Technology Epidemiological Research Ethics Committee and conducted in accordance with the Code of Ethics of the World Medical Association (Declaration of Helsinki).

### Materials

**Figure 1.**
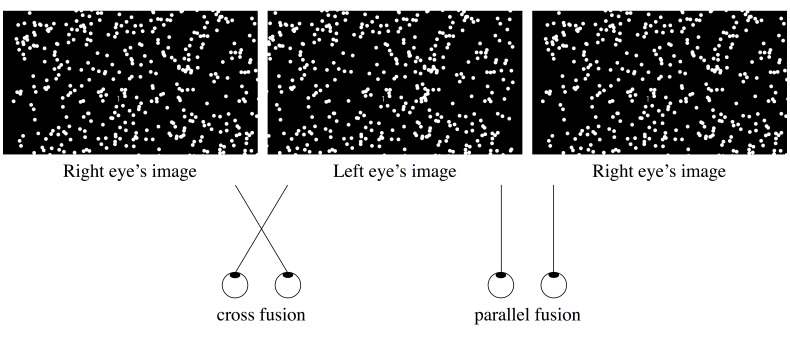
Stimulus configuration used in the experiment: Left and center panels are for cross fusion, and center and right panels are for parallel fusion. By means of free-fusing, both cross and parallel fusers can make an impression on the 3D structure of the stimulus. The numeral “1” indicates the target (presented near the center).

**Figure 2.**
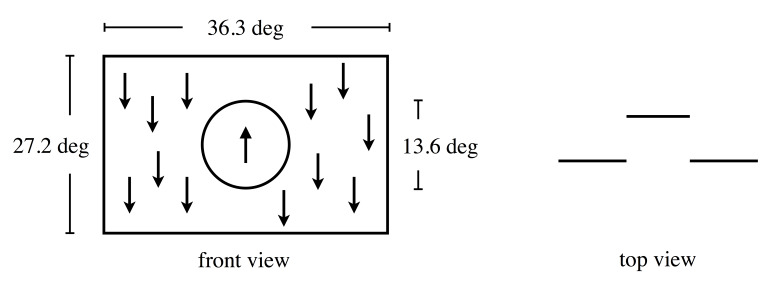
Schematic illustration of stimulus: Left and right panels, respectively, show front and top view of stimuli. Arrows show direction of motion. Circle (not actually presented) show border of area where dots were presented. Central and peripheral areas have different depths.

Figure 1 displays an example of stimuli. Left and right panels are for cross fusion, and center and right panels are for parallel fusion. The stimulus for the left-eye image was drawn in red and viewed through a red filter; the stimulus for the right-eye image was drawn in blue and viewed through a blue filter (the anaglyph technique). Background luminance was 0.01 cd/m^2^. The motion stimulus consisted of randomly positioned moving dots, with a size, velocity, and density of 0.8 deg, 31.0 deg/s, and 0.4 dots/deg^2^, respectively. Luminance of a red dot for the left eye was 8.1 cd/m^2^, and of a blue dot for the right eye was 3.1 cd/m^2^. The difference in luminance between dots for the left and right eyes did not affect stereopsis. Figure 2 illustrates the stimulus schematically. The central stimulus was circular with a diameter of 13.6 deg and presented at the display’s center (the circular line shown in Figure 2 here was not actually presented). The peripheral stimulus was presented in the rest of the display (36.3 × 27.2 deg). The central stimulus and the peripheral stimulus were presented simultaneously. Dots in each stimulus area moved vertically, and areas’ motion directions were always opposite each other (upward and downward). The reason to use vertical motion was to facilitate the horizontal binocular fusion to the stimulus. Because the stimulus had horizontal disparity, the effort for binocular fusion sometimes produced horizontal eye movement similar to OKN. One of the two areas was presented on the display plane (visual distance 57.0 cm), and the other was presented on the plane with 4° (front) or −4° (behind) of disparity with respect to the display plane, corresponding to the theoretical distance of 35.6 cm or 143.8 cm, respectively, when the inter-ocular distance was 6.6 cm. Observers were instructed to confine their vergence to the plane with 4° or −4° of disparity. We used such a large disparity because the two different depth planes were fused when disparity of stimuli was small (Panum’s fusional area [14, 15]). The target used to maintain attention on the instructed depth plane was either “0” or “1.” The target’s size, velocity, direction, and depth were the same as those of the attended plane’s stimulus dots. Dots and the target of the central and peripheral areas disappeared at the areas’ borders.

### Procedure

**Figure 3.**
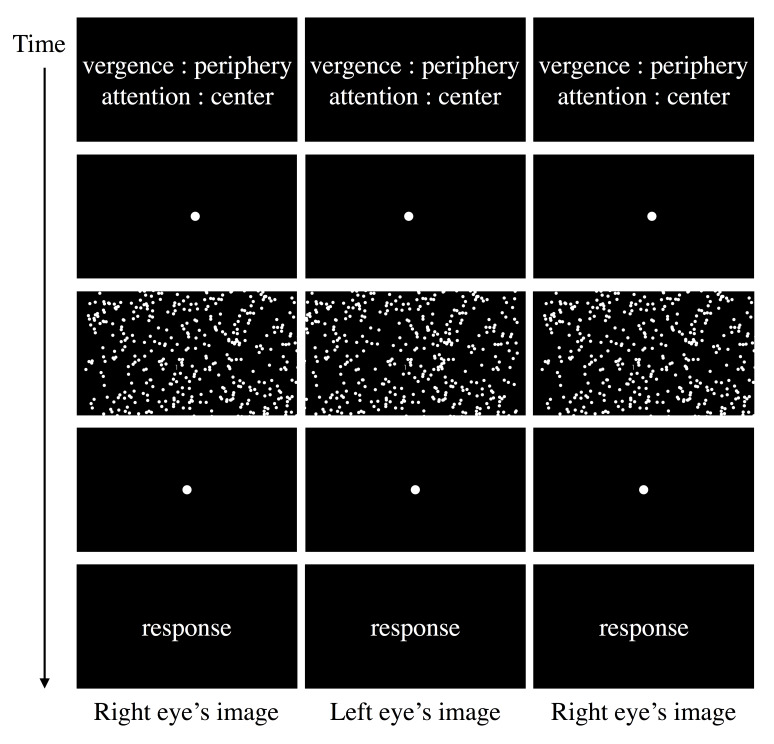
Time course of stimulus presentation in a trial: Left and center panels are for cross fusion, and center and right panels are for parallel fusion. By means of free-fusing, both cross and parallel fusers can make an impression of the 3D structure of the stimulus. The observer attended to the motion stimulus while maintaining vergence at the different (or same) depth plane from attended plane on the anteroposterior axis at the center of stimulus.

Figure 3 displays the time course of stimuli presentation for one trial. In one trial, first, the observer was instructed to which plane to direct attention and vergence. Following the observer’s button press, a pair of dots—one for the left and one for the right eye—were presented. Then, the observer fused them to perceive one dot on the depth plane to which vergence was directed. After the observer fused the two dots, a button press caused stimuli to appear. The observer attended to the instructed motion stimulus while maintaining vergence on the instructed depth plane. During motion stimuli presentation, the observer directed the eyes to the central area. No fixation point was presented, and the duration of stimulus presentation was 3.6 s. In a trial, the target appeared once for 0.8 s in the attended area at randomly decided timing, 1.6–2.5 s after stimulus onset. Each dot appeared at the edge of the stimulus motion area and then moved continually to the area’s other edge. The target disappeared when it exceeded the center circle and the peripheral area’s boundary. A dot with zero disparity appeared for 2.5 s after presentation of a test stimulus. Then, the observer fixated the dot and responded with the target numeral (0 or 1) presented (subjectively). Response time was unlimited, and the observer received feedback. After the response, a button press launched the next trial.

**Figure 4.**
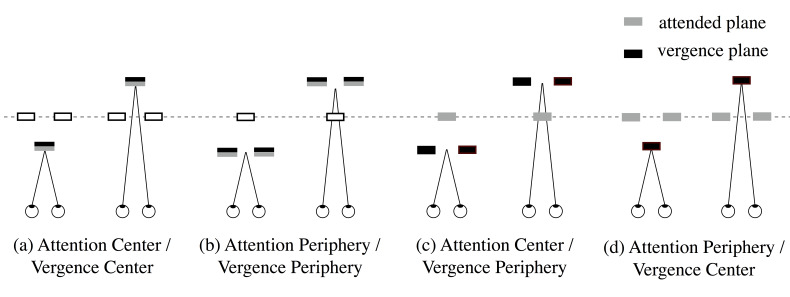
Schematic illustration of stimulus in each condition. All panels show top view. Gray and black squares, respectively, show depth planes where attention was paid and vergence was directed. Dotted lines show the plane of display. See texts for the details.

Figure 4 illustrates a schematic of experimental conditions, i.e., four conditions of vergence and attention: (a) to attend and direct vergence to the center of the stimulus (Attention Center/Vergence Center [ACVC], Figure 4a); (b) to attend and direct vergence to the periphery (Attention Periphery/Vergence Periphery [APVP], Figure 4b); (c) to attend to the center and to direct vergence to the periphery (Attention Center/Vergence Periphery [ACVP], Figure 4c); and (d) to attend to the periphery and to direct vergence to the center (Attention Periphery/Vergence Center [APVC], Figure 4d). These conditions were conducted in separate blocks, and their order differed among observers. Two conditions of vergence (±4°) and two conditions of motion direction (upward and downward) to which to attend were randomized within one block. Each condition of attended and vergence plane was repeated three times for each observer, and each observer took four blocks of different conditions, completing 48 trials.

### Apparatus & analysis

Observers sat in a dark room with their heads fixed on a chin rest, viewing a CRT monitor (GDM F500R, SONY, 1400 × 1050 pixels, 36.3 × 27.2 deg) from a distance of 57 cm. They observed the stimulus while wearing glasses with a red filter for the left eye and a blue filter for the right eye. Stimuli were produced and presented using a PC (MacBook Pro, Apple) and MATLAB (MathWorks) with Psychophysics Toolbox extensions [16, 17, 18]. Observers responded using a numeric keyboard.

Binocular eye positions were recorded with an EyeLink CL (SR Research), a video-based eye tracker, and sampling data with 1000 Hz. Because it has been reported that the contact lenses slip on the eye during and before/after blinks [19], the data during that period is unreliable. Data for 200 ms around eye blinks were excluded from analysis to reduce the noises due to measurement and blinking itself. Peaks at the slow phase and fast phases’ transition points were detected using the “findpeaks” function in MATLAB, which finds local maxima in the data with some parameters. To find relevant peaks corresponding to saccades from the data including noise in the system or ocular tremor, we analyzed the peaks that dropped off on both sides by at least 0.1 deg relative eye position by setting a parameter of “findpeaks” function. OKN frequency was calculated by dividing the number of peaks in one trial by the duration of stimulus presentation (3.6 s). Velocities before and after the peaks were calculated using data for 50 ms and compared. Since it has been reported that the velocity of the fast phase requires no less than 10 deg/s [20], if at least one of the velocities was no less than 10 deg/s, the peak was defined as the point of phase transition in an OKN and the faster velocity of the two phases was defined as a velocity of fast phase. Slow phase velocity was calculated by averaging each trial’s velocities. Next, gains were averaged over three repetitive trials under each condition for each observer. Each OKN’s gain was defined as a ratio of slow phase velocity to stimulus velocity (31.0 deg/s), and the gain was defined as zero when the OKN frequency in the trial was zero. Gains corresponding to motion in attended and non-attended fields were calculated separately. Horizontal vergence was obtained from the two eyes’ visual direction. The interocular distance was assumed to be 6.6 cm. On the basis of the vergence angle to the display distance (6.64°), positive and negative vergence angles were defined as convergence and divergence, respectively.

## Results

**Figure 5.**
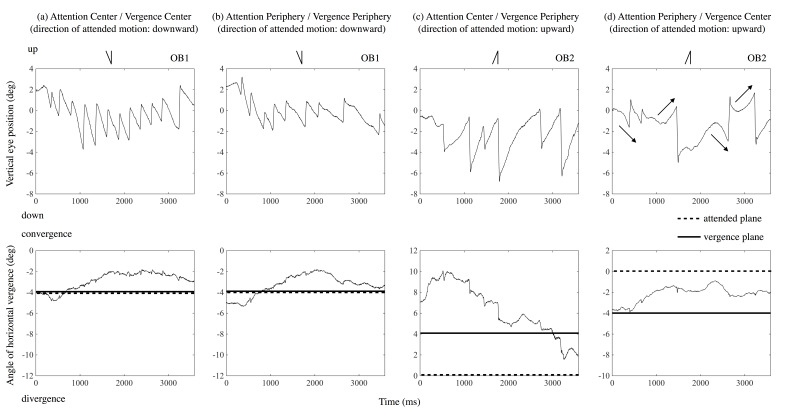
Trace of eye position in a trial for two observers (OB1 and OB2): The upper four panels depict results of vertical eye position. The lower four panels depict results of horizontal vergence. The line drawing inserted above each panel presents a schematic representation of OKN corresponding to the attended motion in the condition. Horizontal lines in the lower panel show the disparity of the plane to which vergence was instructed to direct, and dotted lines show that to which attention was instructed to direct.

Figure 5 shows tracings of eye position for two naïve observers during one trial as examples. Each panel presents: (a) the result of the ACVC condition (attention and vergence directed to the center); (b) APVP condition (attention and vergence directed to the periphery); (c) ACVP condition (attention directed to the center, and vergence to the periphery); or (d) APVC condition (attention directed to the periphery and vergence to the center), respectively. In parentheses under the condition in the figure, the direction of attended motion is noted. The upper panel’s vertical axis presents the eye’s vertical position (deg), signed positive in the display’s upper side. The horizontal axis presents time (ms) from stimulus onset. The lower panel’s vertical axis presents the vergence angle relative to the display (deg), signed positive when eyes converged and negative when they diverged. The solid line shows the disparity of the plane to which eyes were directed, and the dotted line shows the disparity of the plane to which attention was directed. Line drawings inserted in each panel’s upper part present the predicted OKN’s shape, corresponding to the attended motion’s direction for each motion condition. For example, when observers attended to upward motion, the eye’s position was predicted to move upward slowly to pursue the motion of the stimulus and then move quickly downward. The left two and the right two panels show Observer 1 and Observer 2’s results, respectively.

As Figure 5a’s upper panel shows, the eye moved downward slowly to follow the dots and then moved upward quickly when the observer attended to the downward motion in the central area and confined vergence to the same plane. Eye movement was OKN corresponding to the central (attended) motion. Figure 5a’s lower panel shows that the observer confined vergence mostly to the plane with −4° of disparity immediately after the test stimulus was presented, and then vergence shifted slightly to the plane of display (0° of disparity) when the observer was instructed to confine vergence to the plane with −4° of disparity. Similarly, in Figure 5b’s upper panel, the eye moved downward slowly and then moved back quickly when the observer attended to the downward motion in the peripheral area and confined vergence to the plane. Eye movement was OKN corresponding to the peripheral (attended) motion. Figure 5b’s lower panel shows that when the observer confined vergence to the plane with −4° of disparity, vergence varied around −4° of disparity. These OKN results follow those of previous studies [11, 12].

Similarly, in Figure 5c’s upper panel, the eye moved up slowly and then moved down quickly when the observer attended to the upward motion in the central area and confined vergence to the plane of different depth in the peripheral area. Eye movement was OKN corresponding to the attended motion. Figure 5c’s lower panel shows that when the observer was instructed to confine vergence to the plane with 4° of disparity, the observer confined vergence to a position of about 8° of disparity immediately after the test stimulus was presented, and then, with time, vergence shifted to the plane with 4° of disparity. In Figure 5d’s upper panel, however, the eye sometimes moved down slowly and then moved up quickly (the first and third arrows in Figure 5d) when the observer attended the upward motion in the peripheral area and confined vergence to the plane of different depth in the central area. Eye movement was OKN corresponding to motion in the plane to which vergence was confined. Conversely, OKN corresponding to the attended motion also occurred (the second and forth arrows in Figure 5d). In Figure 5d’s lower panel, when the observer was instructed to confine vergence to the plane with −4° of disparity, the observer did so, to a position around −4° of disparity immediately after test stimulus presentation, and then, vergence shifted to a position of about −2° of disparity.

**Figure 6.**
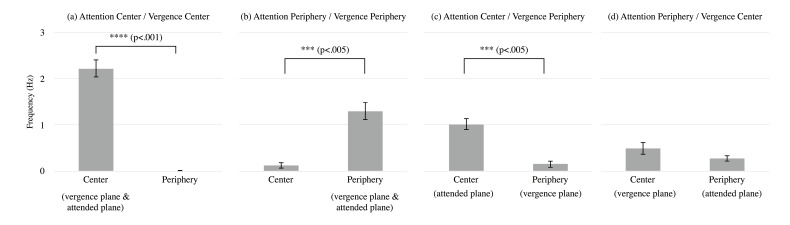
Mean frequency of OKN in the experiment for each condition of directed vergence and attention: The horizontal axis presents the position of stimulus motion to which OKN corresponded, and the parentheses under that indicate instruction for observers. The vertical axis presents the frequency of OKN corresponding to the motion of each area. Error bars show ± 1 SEM.

To clarify the results’ trend, we calculated OKN frequencies corresponding to motion directions of attended and non-attended planes for each trial. In Figure 5a’s

upper panel, for example, the attended motion’s direction was downward, and nystagmus corresponding to the motion occurred 12 times during a trial. We calculated the frequency of OKN for 1 sec and used that as an index, i.e., 3.33 Hz (12/3.6 s). Conversely, the non-attended motion’s direction was upward, and corresponding nystagmus did not occur. Therefore, the OKN frequency was 0. Averaged OKN frequencies for vergence 4° condition in ACVC, APVP, ACVP, and APVC were 2.407 (0.406), 1.310 (0.406), 1.184 (0.364), and 0.245 (0.164), respectively (the value in parentheses shows standard deviation). Similarly, averaged OKN frequencies for vergence −4° condition were 2.037 (0.682), 1.296 (0.536), 0.853 (0.406), and 0.311 (0.221). Averaged OKN frequencies for the upward condition were 1.872 (0.554), 1.303 (0.720), 0.926 (0.518), and 0.238 (0.286). Averaged OKN frequencies for the downward condition were 2.573 (0.606), 1.303 (0.413), 1.111 (0.541), and 0.317 (0.120). For all conditions, results of different depths (±4°) and of motion directions (upward and downward) were averaged because the OKN frequency did not significantly differ for different depths (main effects of *condition*; *F*(3,18) = 32.262, *p* < .001, main effects of *depth* ; *F*(1,6) = 2.612, *p* > .10, interaction of *condition* vs. *depth*; *F*(3,18) = 1.903, *p* > .10), and motion directions (main effects of *condition*; *F*(3,18) = 32.262, *p* < .001, main effects of *direction* ; *F*(1,6) = 1.116, *p* > .10, interaction of *condition* vs. *direction*; *F*(3,18) = 3.107, *p* > .05) for each observer. Averaged results across observers are shown in Figure 6. Each panel presents the result for each combination of conditions of vergence and attended plane as shown in Figure 4. The horizontal axis presents the position of stimulus motion and of instructed vergence and attention in parentheses. The vertical axis presents OKN frequency corresponding to each area’s motion. Error bars show ± SEM.

The result in Figure 6a clearly shows that OKN frequency corresponding to central motion was significantly higher than that corresponding to peripheral motion when both attention and vergence were directed to the central area (*F*(1,6) = 129.380, *p* < .001). This result was expected due to results from previous studies. Similarly, the result in Figure 6b shows that OKN frequency corresponding to peripheral motion was significantly higher than that corresponding to central motion when both attention and vergence were directed to the peripheral area (*F*(1,6) = 25.032, *p* < .005). The result in Figure 6c shows that OKN frequency corresponding to central

motion was significantly higher than that corresponding to peripheral motion when attention was directed to the central area and vergence was directed to the peripheral area’s depth plane (*F*(1,6) = 29.841, *p* < .005). The result in Figure 6d shows, however, that OKN frequency corresponding to peripheral motion and to central motion did not significantly differ when attention was directed to the peripheral area and vergence to the central area ( *F*(1,6) = 1.404, *p* > .10).

**Figure 7.**
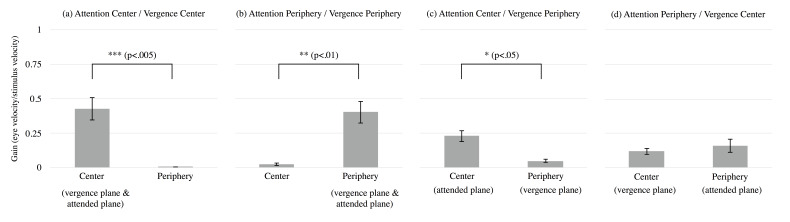
Mean gain of OKN in the experiment for each condition of vergence and attention instructed to direct: The vertical axis presents the gain of OKN corresponding to the motion of each area. Other aspects of the figure are the same as those in Fig 6.

To verify the significance of results of OKN frequency corresponding to the attended motion mentioned above, a one-way ANOVA was performed on data for the four conditions. The main effect of *condition* was significant for frequency ( *F*(3,18) = 32.262, *p* < .001). Multiple comparison tests using Ryan’s method (α = 0.05) showed that differences between any combinations of results were significant, except for that between results of the APVP and the ACVP conditions ( *p* > .10). As with analytical results on OKN frequency, we calculated the OKN gain corresponding to attended motion and non-attended motion in each trial. For example, in Figure 5c’s upper panel, the direction of non-attended motion was downward, and corresponding nystagmus did not occur. Therefore, the OKN gain corresponding to non-attended motion in this trial was 0. Conversely, the direction of attended motion was upward, and corresponding nystagmus occurred six times. Therefore, the average gain across six OKNs, 0.22, was used as the OKN gain corresponding to this trial’s attended motion. Averaged OKN gains for vergence 4° condition in ACVC, APVP, ACVP, and APVC were 0.416 (0.165), 0.400 (0.244), 0.258 (0.088), and 0.158 (0.128), respectively (the value in parentheses shows standard deviation). Similarly, averaged OKN gains for vergence −4° condition were 0.425 (0.231), 0.403 (0.176), 0.198 (0.128), and 0.164 (0.120). Averaged OKN gains for the upward condition were 0.395 (0.196), 0.3424 (0.266), 0.224 (0.118), and 0.194 (0.201). Averaged OKN gains for the downward condition were 0.459 (0.274), 0.379 (0.206), 0.232 (0.118), and 0.127 (0.065). Results of different depths (± 4°) and motion directions (upward and downward) were averaged in each condition because the OKN gain did not differ significantly for different depths (main effects of *condition*; *F*(3,18) = 9.388, *p* < .001, main effects of *depth* ; *F*(1,6) = 0.509, *p* > .10, interaction of *condition* vs. *depth*; *F*(3,18) = 0.678, *p* > .10) and motion directions (main effects of *condition*; *F*(3,18) = 9.404, *p* < .001, main effects of *direction* ; *F*(1,6) = 0.025, *p* > .10, interaction of *condition* vs. *direction*; *F*(3,18) = 0.845, *p* > .10) in each condition. Average results of gain across observers are shown in Figure 7. The horizontal axis is the same as in Figure 6. The vertical axis shows the gain (slow phase velocity/stimulus velocity) of OKN corresponding to motion of the central or peripheral areas. Error bars show ± SEM. The result in Figure 7a shows that the OKN gain corresponding to central motion was significantly higher than that corresponding to peripheral motion when both attention and vergence were directed to the central area ( *F*(1,6) = 27.134, *p* < .005). The result in Figure 7b shows that OKN gain corresponding to peripheral motion was significantly higher than that corresponding to central motion when both attention and vergence were directed to the peripheral area (*F*(1,6) = 17.588, *p* < .01), although motion in the opposite direction was presented in the central area. These results are consistent with previous studies and qualitatively consistent with present frequency results (Figures 6a and 6b). The result in Figure 7c shows that OKN gain corresponding to central motion was significantly higher than that corresponding to peripheral motion when attention was directed to the center area and vergence was directed to the peripheral area (*F*(1,6) = 11.851, *p* < .05). The result in Figure 7d shows that OKN gain corresponding to peripheral motion did not significantly differ from that corresponding to central motion when attention was directed to the peripheral area and vergence was directed to the central area ( *F*(1,6) = 0.364, *p* > .10). These results are also qualitatively consistent with frequency results (Figures 6c and 6d).

To verify the significance of results of OKN gain corresponding to the attended motion mentioned above, a one-way ANOVA was performed on data for the four conditions. As a result, the main effect of *condition* was significant for frequency ( *F*(3,18) = 9.404, *p* < .001). Multiple comparison tests using Ryan’s method (α = 0.05) showed that differences between any combination of results were significant, except for the combination in which attention and vergence were directed to the same plane (Figures 6a and 6b) and that in which attention and vergence were directed to different planes (Figures 6c and 6d) ( *p* > .10).

Mean results of the vergence angle (deg) and percentage of correct answers for the target detection task (%) in each condition are shown in Table 1. The mean percentage of keeping vergence within ±1 deg of the instructed plane during a trial in conditions of ACVC, APVP, ACVP, and APVC were 38.0 (17.7), 29.6 (18.9), 22.7 (12.2) and 30.3 (14.6) %, respectively (the value in parentheses shows standard deviation). In the table, we also present results of a paired t-test to verify whether the mean vergence angle when confining vergence to 4° and −4° of disparity differed significantly from theoretical values of 4° and −4° respectively. As a result, in all conditions, the mean vergence angle when confining vergence to 4° of disparity (cross disparity) did not differ significantly from the theoretical value of 4°; however, the mean vergence angle when confining vergence to −4° of disparity (uncrossed disparity) did significantly differ from the theoretical value of −4°. This result indicates that the mean vergence to −4° of disparity was not directed to the instructed vergence plane. To test for significance in differences of mean vergence angle in each condition when instructed to direct vergence to −4° of disparity, a one-way ANOVA was performed. The main effect of *condition* was not significant for mean vergence angle (*F*(3,18) = 0.796, *p* > .50). Therefore, we suppose the reason for the difference in the results of OKN frequency and gain in these conditions was caused by directing attention and not by the difference in vergence angle.

**Table 1:** Mean vergence angle, a paired t-test, and accuracy of target detection task in each condition.

	**ACVC**		**APVP**		**ACVP**		**APVC**	
**(a) Vergence condition**	4°	−4°	4°	−4°	4°	−4°	4°	−4°
**(b) Mean angle of vergence (SE)**	3.96° (0.51)	−2.35° (0.34)	4.49° (0.65)	−2.85° (0.19)	4.42° (0.63)	−2.34° (0.21)	4.25° (0.11)	−2.71° (0.48)
**(c) *p* value (t-test): (a) vs (b)**	0.95	0.029*	0.48	0.001*	0.52	0.000*	0.068	0.038*
**(d) Percent correct (SE)**	66.6% (7.7)		69.6% (4.5)		70.2% (4.7)		45.2% (5.0)	

ACVC condition (attention and vergence directed to the center); APVP condition (attention and vergence directed to the periphery); ACVP condition (attention directed to the center, and vergence to the periphery); APVC condition (attention directed to the periphery and vergence to the center).

Mean correct-answer rates to the target detection task in conditions of attended and vergence plane, ACVC, APVP, ACVP, and APVC (Figures 4a, 4b, 4c, and 4d) were 66.6%, 69.6%, 70.2%, and 45.2%, respectively. The correct-answer rate was about 66% in the ACVC condition, which indicates that this task sufficient for participants to maintain their attention until the target was detected. To test for significance in differences of correct-answer mean rates in each condition, a one-way ANOVA was performed. The main effect of *condition* was significant for mean rates of correct answer (*F*(3,18) = 4.845, *p* < .05). Multiple comparison tests using Ryan’s method (α = 0.05) showed that differences between the value in APVC (attention directed to the peripheral area and vergence to the central area) and the value in other conditions differed significantly ( *p* < .05).

## Discussion

In this study, we examined the more essential factors of motion for generating OKN, retinal location, vergence location, or attentional location. For this purpose, we investigated OKN properties when two motions with different directions were presented in central and peripheral visual fields and on different depth planes, while separately manipulating vergence and attention direction. As a result, OKN corresponding to attended motion occurred when the plane of attended motion was the same as the plane of vergence, no matter whether motion was presented in the periphery or the center. This result indicates that retinal location is not essential for generating OKN and is consistent with the previous study [12]. In the condition with attention directed to the center and vergence to the plane of periphery, OKN corresponding to attended motion mainly occurred. However, in the condition with attention directed to the periphery and vergence to the center, OKN corresponding to the motion of the attended plane and of the vergence plane occurred equally. These results indicate that attention is always necessary for OKN’s occurrence but vergence is not necessarily important for OKN’s occurrence.

Analysis of the relationship between the vergence position and OKN frequency indicates that motion in the vergence plane is not essential for OKN to occur. In the condition that the plane of attention was consistent with the plane of vergence, observers exactly confined vergence on the instructed plane when the stimulus had crossed disparity (front), while observers confined vergence in front of the instructed plane when the stimulus had uncrossed disparity (behind). However, OKN frequencies and gains in these conditions did not differ significantly. In addition, in conditions in which attention and vergence were directed to different planes, OKNs corresponding to the attended plane’s motion occurred, but those corresponding to motion on the vergence plane were much less or about the same. These results indicate that vergence is not an essential factor for OKN’s occurrence.

From the present experiment’s results, we presume that attention is the essential factor in producing OKN when motions with different directions are presented at different depths. This is indicated because many OKNs corresponding to attended motion occurred in the ACVP condition (attention directed to the center and vergence to the periphery) (Figure 4c) and in conditions of ACVC and APVP (Figures 4a and 4b). The results that OKN frequency and gain corresponding to attended motion and to motion on the vergence plane did not differ significantly in the APVC condition (attention directed to the periphery and vergence to the center) (Figure 4d) might indicate the importance of motion on the plane of vergence for OKN. However, the accuracy rate of target detection tasks in this condition was much lower (45.2%) than those in other conditions (66.6%, 69.6%, and 70.2%). These results indicate that the magnitude of attention directed to the instructed plane in the APVC condition was weaker than in other conditions. The target detection rates in this condition were likely hindered by the lack of robust OKN. As shown in Figure 5, vergence shifted from the vergence plane to the target plane during a trial. This fact indicates that attention and vergence were not completely separated. Therefore, it would be possible to suppose that the target detection rate was low because the occurrence of OKN decreased and the image of a target was not stabilized on the retina properly.

Results below are also consistent with attention’s magnitude being essential for OKN frequency and gain. OKN gains corresponding to attended motion were lower in conditions in which attended and vergence planes differed than in conditions in which they were the same. This is presumed a decrease in the magnitude of attention to the attended plane due to attention remaining on the plane of vergence. In the previous study, OKN corresponding to motion of the central area occurred when observers confined vergence to the peripheral area [12], although this hardly occurred in this study (Figure 6b). We suppose the reason for the difference is the target detection task’s existence. We also suppose that OKN corresponding to the central motion occurred in the previous study [12] because observers’ attention remained in the central area as well as in the peripheral area. Moreover, OKN corresponding to central motion did not occur in the present study because the target detection task caused steady, focused attention on peripheral motion.

However, we did not deny the possibility that stimulus features have direct effects on OKN frequency and gain. In some conditions, a difference appeared in OKN frequency and gain, but no difference in the target detection task’s accuracy rate, thus indicating this presumption. For example, in conditions ACVC and APVP (attention and vergence directed to the same plane), no significant difference appeared in the target detection task’s accuracy rate although significant difference appeared in OKN frequency. In addition, in conditions in which attended and vergence plane were inconsistent, OKN gains were lower than in conditions in which attended and vergence plane were consistent. As mentioned in the previous study [13], the reason for the difference is linkage between the optokinetic system and the stereoscopic system. The OKN gain in animals with stereoscopic vision was higher than those without it because the stereoscopic signal routed through the visual cortex supplements direct inputs from the retina to the pretectum [21, 22, 23]. The OKN gain would decrease in conditions in which attended and vergence plane were inconsistent because supplemental cortical inputs to subcortical mechanisms controlling OKN decreased due to the diplopic image of attended motion.

Attention can be voluntarily separated from the position of the gaze and vergence although attention is normally linked to them. However, their connection to attention is enhanced by gazing and directing vergence together. In such a case, observers have difficulty directing attention to a different position and depth from the point of gaze and vergence. OKN corresponding to attended motion occurred when motion stimuli with different directions were presented at central and peripheral visual fields on a planar surface [11] or when attention was directed to the central field and vergence was directed to the peripheral plane at a different depth from the center. However, OKNs corresponding to the attended motion were weak in the APVC condition, probably due to the decrease in attentional magnitude to the peripheral area because central vision and binocular fusing were not combined. In summary, separating attention from the central area is not difficult, but separating attention from a binocularly fused image on the central area is quite difficult. Certainly, since physical factors such as stimulus velocity, size, and motion direction (orthogonal directions) are related to OKN [5], the relationship between these and attention should also be considered and such an investigation is needed.

## Conclusion

In this study, we investigated whether OKN corresponding to attended motion occurred when two motions in different directions were presented in central and peripheral visual fields separated by depth. As a result, in conditions in which attention and vergence were directed to the same plane, OKN corresponding to motion on the plane of attention and vergence occurred regardless of the motion’s presentation position. In the ACVP condition (attention directed to the center and vergence to the periphery), OKN corresponding to the attended motion occurred. In the APVC condition (attention directed to the periphery and vergence to the center), however, OKNs corresponding to motions on the attention plane and on the vergence plane occurred. Analysis of horizontal vergence and the target detection task’s accuracy rate during the trial indicated that the motion of the attended position, rather than that of the vergence position or that on the central visual field, is essential for occurrence of OKN. The relationship between OKN frequency and gain and the accuracy rate of target detection task during the trial is consistent with the idea that magnitude of attention is essential for properties of OKN.

Recently, several reports have demonstrated that visual attention relates to pupillary light reflex [24, 25, 26], micro-saccades [27, 28], and vergence eye movements [29, 30]. We suppose it possible to predict the directed area of attention based on OKN direction when areas of motion have different directions in the visual field. By using this method to predict attentional location in a visual stimulus with various directions of motion from OKN, it would be possible to know the location to which a driver is attending (to up or down) in the optical flow, for example. In addition, predicting attentional state and position more accurately by combining knowledge from the present study and previous studies’ findings would be possible. To realize such a system, we need to ascertain the relationship between attention and OKN, pupillary response, micro-saccades and other eye movements in more complex situations in real scenes.

## Ethics and Conflict of Interest

The authors declare that the contents of the article are in agreement with the ethics described in http://biblio.unibe.ch/portale/elibrary/BOP/jemr/ethics.html and that there is no conflict of interest regarding the publication of this paper.

## Acknowledgements

We appreciate the volunteers for participating in this study and anonymous reviewers for their valuable comments and suggestions, which improved the quality of the paper.
